# A three-stage assembly program governing pancreatic, plasma, pituitary, and bone secretory cell differentiation: A strategy to augment protein delivery

**DOI:** 10.1016/j.jbc.2025.110562

**Published:** 2025-08-05

**Authors:** Joseph P. Bidwell, Alexander G. Robling, Ronald C. Wek

**Affiliations:** 1 Department of Anatomy, Cell Biology & Physiology, Indiana University School of Medicine (IUSM), Indianapolis, Indiana, USA; 2 Indiana Center for Musculoskeletal Health, IUSM, Indianapolis, Indiana, USA; 3 Richard L. Roudebush VA Medical Center, Indianapolis, Indiana, USA; 4 Department of Biochemistry and Molecular Biology, IUSM, Indianapolis, Indiana, USA

**Keywords:** Bone, cell differentiation, integrated stress response, lineage-determining factor, scaling factor, osteoporosis, protein secretion, unfolded protein response

## Abstract

Defective secretory cell function underlies many diseases, and recent therapeutic strategies have focused on enhancing protein synthesis and delivery by targeting the secretory machinery of mature cells. However, mature differentiated cells appear to have intrinsic limits to their secretory capacity. In this review, we propose new strategies for engineering these cells to overcome these limits on secretion. The integrated stress response (ISR) and the related unfolded protein response (UPR) are stress adaptation systems that modulate transcriptional and translational programs of gene expression. These programs drive remodeling of cellular architecture to boost protein production and trafficking but also play critical roles in the differentiation of secretory cells. This dual function suggests that the limits of the secretory capacity of mature cells are pre-programmed during development. A potentially more effective therapeutic approach to expand protein secretion may lie in reprogramming the secretory capacity early in differentiation. Two additional transcriptional programs work in concert with the ISR and UPR to shape differentiated cell identities, their secretory outputs, and production capacity. The first involves lineage-determining transcription factors that define both cell type and secretory products. The second involves “scaling factors” that set the magnitude of the cell’s protein synthesis and secretion capacity. We explore the mechanisms by which these three programs—lineage specification, scaling, and stress adaptation—interact to define and potentially enhance secretory capacity. We will illustrate this integrated model across several secretory cell types, including pancreatic, plasma, pituitary, and bone secretory cells, with a focus on applications to enhance therapeutic outcomes in osteoporosis.

Professional secretory cells are functionally defined by their proficiency to synthesize, process, and release vast quantities of protein products (1). These cells undergo a spectacular metamorphosis in architecture from progenitor to mature secretory cell, eliciting enlargement of the secretory machinery including the endoplasmic reticulum (ER) and the Golgi apparatus, accompanied by an increase in the protein synthesis apparatus (2, 3, 4, 5, 6, 7, 8). However, all secretory cells have limits to their output, and many diseases stem at least in part from cell constraints on protein secretion (9, 10, 11). Recent strategies to enhance protein output by manipulating the secretory machinery of mature cells have shown limited success, likely due to the built-in limits on their secretory capacity (9, 10, 11, 12, 13, 14, 15, 16, 17). In this review, we propose an alternative strategy: reprogramming secretory progenitor cells to bypass these intrinsic constraints and unlock enhanced secretory potential.

Secretion is modulated by environmental and physiological cues, sensed in part by the unfolded protein response (UPR) and the related integrated stress response (ISR) (18, 19, 20, 21). These pathways coordinate the expansion of secretory organelles and the protein synthesis and folding machinery to support the activity of fully differentiated cells. Evidence suggests that the capacity for protein synthesis and secretion is largely established early in differentiation, guided by the integration of three transcriptional programs (Fig. 1) (22, 23, 24, 25, 26, 27, 28, 29). The first is driven by lineage-defining transcription factors (TF^Lineage^) that determine cell identity and specify the secretory product. The second involves "scaling factors" (TF^Scaling^ f^actor^) that shape the epigenetic and transcriptional landscape to define the upper limits of output in the mature cell (29). These scaling factors modulate transcriptional activity but rely on initiation cues from lineage-specific regulators and stress-responsive signals, such as those from the ISR and UPR. The third program comprises the transcriptional responses directly mediated by the ISR and UPR pathways themselves (TF^ISR/UPR^). These three programs are not fully independent; instead, they can converge at multiple levels. They can share target genes, and some transcription factors participate in more than one program; for example, an ISR/UPR-responsive factor may also function as a scaling factor (22, 30, 31).

**Figure 1:**
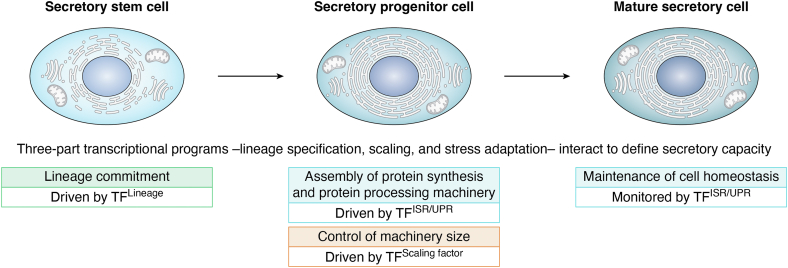
**The three-part transcriptional program for assembling a secretory cell.** The ISR and UPR provide a sensing system that re-directs or modifies secretory activity in reaction to changing demands or environmental insults. Recent studies indicate that activation of these programs is not reserved as a response to stress but is integral to the differentiation of professional secretory cells. Moreover, the protein production and processing capacity of the mature cell is programmed before it becomes secretory. Evidence suggests differentiation of these cells requires three kinds of transcriptional programs that function in concert. One group of transcription factors are lineage-directing and specify both cell type and secretory products (TF^Lineage^). The second are launched by the ISR/UPR that direct the marked changes in cellular architecture, specifically the expansion of the protein production and secretory machinery (TF^ISR/UPR^). The third are the ‘scaling factors’ that fix the capacity of this machinery (TF^Scaling factor^).

The ISR and UPR are under active investigation as therapeutic targets, with several compounds developed as research tools and a few progressing into clinical trials (9, 10, 11, 12, 13, 14, 15, 16, 17). However, these strategies face notable limitations. Most current approaches focus on manipulating fully differentiated cells, which inherently possess fixed limits on their secretory capacity. Forcing these cells beyond their natural thresholds can lead to maladaptive ISR/UPR activation and trigger apoptosis (18, 32). Can secretory capacity be reprogrammed during the development of adult stem or progenitor cells? To achieve maximal output from professional secretory cells, it is essential to appreciate how the ISR/UPR pathways interact with lineage-specifying and scaling factors early in the differentiation process. This developmental integration may provide a more sustainable and adaptable route to enhancing secretory function in health and disease.

This review explores the differentiation of professional secretory cells, focusing on the mechanisms by which lineage is specified, the secretory machinery is expanded, and functional capacity is established. We begin by examining the roles of the ISR and UPR in coordinating protein synthesis and secretion and then discuss how these pathways intersect with lineage-determining transcription factors and scaling factors that define the secretory output of mature cells. Throughout the review, we highlight examples from diverse secretory tissues—including the exocrine pancreas, immune plasma cells, and the pituitary gland. In the final section, we narrow our focus to the role of these three regulatory programs in osteoblast differentiation and bone formation, considering their relevance to the efficacy of current osteoporosis therapies. Targeting ISR/UPR-related mechanisms during early stages of differentiation may offer more durable and effective treatment strategies for a wide range of secretory disorders than current approaches that act on fully differentiated cells.

## The ISR/UPR, a primer

**Figure 2:**
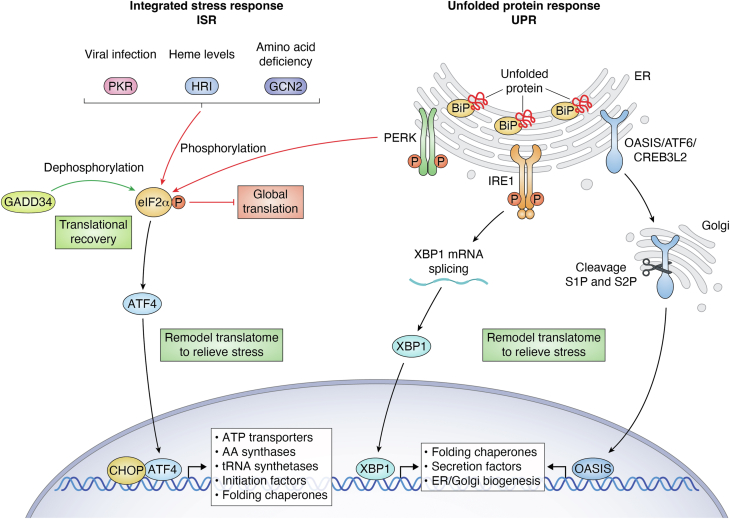
**The ISR/UPR BASICS: Various cellular stresses activate the ISR by mobilizing one of four protein kinases: GCN2, PERK, PKR, and HRI.** Each of these kinases phosphorylate the alpha subunit of eIF2. Phosphorylation of eIF2 causes a reduction in global translation while simultaneously promoting the preferential translation of ISR-specific mRNAs, such as ATF4. ATF4 is a key transcription factor in the ISR, increasing the expression of hundreds of genes that function in stress adaptation. Global translation resumes upon the dephosphorylation of eIF2 by the constitutively expressed CReP (not shown) and ISR-inducible GADD34, both of which target protein phosphatase type 1. When the protein folding demands exceed the processing capacity of the ER, the accumulating unfolded proteins induces the UPR. The UPR consists of ER stress sensors, PERK, IRE1, and OASIS (also ATF6 and CREB3L2), all situated in this organelle. Activation of these sensory proteins occurs upon accumulation of unfolded protein in the ER. It is suggested that unfolded proteins bind to the molecular chaperone BiP, releasing its inhibitory association with the ER sensors. Additionally, unfolded proteins may directly engage with sensory proteins, triggering their activation. PERK also functions in the ISR and phosphorylates eIF2 as described. IRE1 has both a serine/threonine protein kinase domain and an endoribonuclease domain that facilitates splicing of Xbp1 mRNA, leading to the synthesis of an active transcription factor that enhances expression of stress adaptive genes. Activation of OASIS, ATF6, and CREB3L2 triggers their translocation to the Golgi complex where they are proteolytically cleaved into active transcription factors. Both the ISR and the UPR act to enhance protein production, processing, and secretion.

There is accumulating research supporting the idea that the machinery of a professional secretory cell is built, and its capacity is programmed, by the combined action of the physiological or non-stressed versions of the ISR/UPR (28, 33). Many excellent reviews have described the ISR and UPR in depth, so here we will only focus on key aspects of these pathways (18, 19, 20, 32, 34, 35). The ISR coordinates the amounts of protein synthesis in cells to environmental and physiological conditions. For example, accumulating unfolded proteins in the ER or increasing demand for amino acids can activate a family of sensory ISR protein kinases that phosphorylate the alpha subunit of eIF2 at Ser51, reducing its ability to deliver initiator tRNA to the translational machinery. The resulting lowered protein synthesis conserves energy and nutrients and helps coordinate reprogramming of gene expression towards optimal cell adaptation. To facilitate the reprogramming, eIF2 phosphorylation can also direct preferential translation of specific gene transcripts, such as ATF4 that directs the transcription of genes involved in restoration of cell homoeostasis and in ISR feedback control (20, 36, 37). In this way, the ISR helps to direct both translational and transcriptional modes of gene expression. This pathway is self-limiting *via* dephosphorylation of eIF2 by the upregulation of the protein phosphatase 1 (PP1) regulatory subunit GADD34 (PPP1R15A) (18, 38, 39). Similarly, CReP (PPP1R15B) confers PP1 specificity for phosphorylated eIF2. Both GADD34 and CReP are critical for translational recovery (18, 20, 40, 41, 42) (Fig. 2).

One of four eIF2 kinases function as sensory proteins in the ISR and each monitor different perturbations in cells (Fig. 2). These four kinases include the PKR-like ER kinase, PERK (EIF2AK3) that is activated in response to unfolded proteins in the ER and General control nonderepressible 2, GCN2 (EIF2AK4), which responds to uncharged tRNAs that are elevated in the cell when free amino acids are limited (34). Heme-regulated inhibitor (HRI) acts as a key sensor for heme deficiency and mitochondrial stress (43) and double-stranded RNA-dependent protein kinase (PKR) participates in viral defense (44) In this way, phosphorylation of eIF2 and its coordinated translational and transcriptional modes of gene expression respond to different stress conditions. It is noted that while select environmental stresses are potent activators of each of these eIF2 kinases, physiological stresses can also trigger their activation. Depending on the stress arrangement, the ISR can function in conjunction with other adaptive pathways, such as those involving protein secretion, to best tailor the adaptive gene expression for the stress condition and cell type.

Secreted proteins comprise about 13% of all human protein-coding genes (45). In a recent study to profile these proteins, they started with a bioinformatics-based definition of the secretome and grouped the identified proteins into three main categories: (i) blood proteins, (ii) locally secreted proteins, and (iii) intracellular proteins (45). Although the inclusion of intracellular proteins may seem counterintuitive, it reflects the idea that many proteins initially targeted to the endoplasmic reticulum (ER) are ultimately directed to intracellular destinations—such as lysosomes, or retained within the ER or Golgi—rather than being secreted outside the cell. The oncoming massive increase in secretory protein production can put demands on the post-translational processing machinery of the cell, specifically the ER and Golgi organelles (33). Secretory and membrane proteins enter the ER for folding and initial post-translational modifications. ER protein-folding chaperones, including BiP (HSPA5), assist in coaxing the secretory proteins into their proper 3D conformation (46). From there, proteins slated for the secretory path are delivered to the Golgi for further processing and are ultimately destined to be secreted from the cell, or to the membranes of the cell and its organelles.

The UPR is the primary mechanism for maintaining the smooth operation of the protein processing machinery or expanding it to meet the anticipated needs of the mature secretory cell (18, 19). If the gridlock in processing and secretion cannot be resolved, the UPR instead activates apoptosis. Three ER proteins can sense unfolded polypeptides in the ER lumen and upon activation by ER stress launch signaling cascades that activate distinct but overlapping translational and transcriptional programs for meeting the increased demand for protein processing (47) (Fig. 2). The three sensors include PERK, which also functions in the ISR, along with IRE1 (ERN1) and ATF6. Each of the UPR sensors is a transmembrane protein with a portion in the ER lumen that can directly or indirectly sense unfolded proteins, and a cytoplasmic portion that functions in UPR signaling. As highlighted above, upon activation PERK phosphorylates eIF2, resulting in transient lowering of translation that relieves the nascent protein load into the ER. IRE1 is a serine/threonine-protein kinase and endoribonuclease. The latter helps to facilitate the splicing of a 26-nucleotide segment from the mRNA encoding the transcription factor XBP1, leading to translation of an active transcription factor for expression of genes involved in ER protein translocation, protein folding and secretion, and the elimination of misfolded proteins. Upon ER stress, ATF6 is released from the ER to Golgi, where it undergoes regulated intramembrane proteolysis (RIP). Two Golgi-localized proteases cleave this protein to release the NH2-terminal portion of ATF6 to enter the nucleus where it can drive the expression of UPR genes that contribute to the folding and trafficking of secretory proteins. The ER-resident OASIS (CREB3L1), and CREB3L2 (BBF2H7) are additional UPR transcription factors that are also subjected to RIP (47) (Fig. 2). Collectively, these transcription factors and those triggered by the ISR can serve as effectors for programming the transcriptome for optimized synthesis, folding, and secretion of proteins.

## Setting secretory capacity from the start

**Figure 3:**
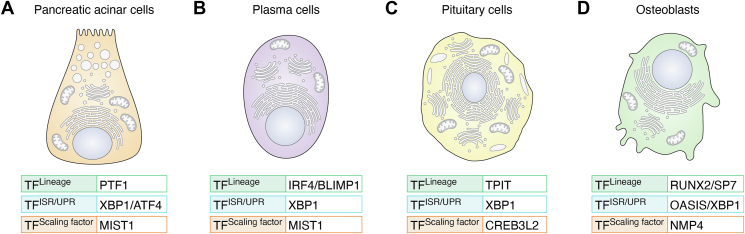
**The three-part transcriptional program for assembling professional secretory cells.** Four specific examples are indicated. *A*, the development of the pancreatic acinar cell requires PTF1 as a key TF^Lineage^, XBP1 and ATF4 as the TF^ISR/UPR^, and MIST1 serves as the TF^Scaling factor^. *B*, plasma cell development is driven by IRF4 and BLIMP1 (TF^Lineage^), XBP1 (TF^ISR/UPR^), and MIST1 as the TF^Scaling factor^. *C*, the assembly of pituitary cells is primarily driven by the TF^Lineage^ TIPIT, and the TF^ISR/UPR^ XBP1. The UPR protein CREB3L2 multitasks as a TF^Scaling factor^. *D*, building a secretory osteoblast requires RUNX2 and SP7 as TF^Lineage^, OASIS and XBP1 as TF^ISR/UPR^, and NMP4 as the TF^Scaling factor^. Although the listed transcription factors are key drivers of the processes described, other trans-acting proteins are involved in the development of these cells as described for pancreatic acinar cells (204), plasma cells (77), pituitary cells (22), and osteoblasts (153, 205).

Emerging from the studies of professional secretory cell differentiation, the ISR/UPR transcriptional programs work in concert with lineage-directing regulators and scaling factors to delineate the development and processing capacity limits of the mature secretory cell (22, 29, 30, 48). As detailed above, the transcription factors launched by the ‘physiological’ form of the ISR/UPR (indicated as TF^ISR/UPR^ in Fig. 1) build the protein synthesis apparatus, protein processing, and secretory machinery in anticipation of meeting the demands of delivering large secretory loads. Cell fate or lineage commitment transcription factors, indicated as TF^Lineage^ in Figure 1, mandate the identity of the cell and specify its secretory products. Scaling transcription factors, or TF^Scaling factor^ (Fig. 1), ostensibly govern the capacity of the protein production/secretory machinery (29). It is noted that these transcription factors can assume different roles in secretory cell development contingent upon the tissue. We now examine the more detailed features of differentiation or assembly of selected professional secretory cells within the framework of this three-part transcriptional program. The key transcription factors and their organization in the three programs for each of the featured secretory cells are illustrated in Figure 3, *A*–*D*.

### Pancreatic acinar cells

There is coordinated induction of ISR/UPR, lineage-directing and scaling factors that occurs during the development of a secretory cell that governs its type and processing capacity. For example, differentiation of pancreatic acinar cells is driven by this three-part program (Fig. 3*A*). Mature acinar cells display an elaborate ER and Golgi structure (49, 50). The zymogen granules (ZG) of these cells contain digestive enzymes, including lipases, proteases, and enzymes which digest carbohydrates (50, 51), which are released into the ductal system and ultimately the small intestine for processing of food. The program of acinar cell lineage is governed in part by a TF^Lineage^ designated PTF1 (30, 52, 53, 54), a multisubunit complex containing PTF1a and TCF12, both basic helix-loop-helix (bHLH) E-box-binding transcription factors (30, 52, 53, 54). A third member of the PTF1 complex is RBPJ, a trans-acting protein that recognizes TC-box elements and plays a role in cell fate decisions; RBPJ serves as a counterbalance to Notch signaling that can drive differentiation towards ductal and endocrine lineages (55, 56, 57, 58, 59). Transcription activation requires that the PTF1 complex binds to tandem E− and TC-box elements found in many zymogen genes as well as several transcription factor genes crucial for acinar cell development and function (30, 52, 53, 54). As the acinar cells mature, RBPJ is replaced by RBPKL, closely related to RBPJ, further maximizing secretory protein synthesis and their packaging as well as other functions necessary for maintaining the secretory phenotype of mature acinar cells (55).

The ISR/UPR transcription factors XBP1 and ATF4 are also critical for acinar cell differentiation, contributing to the assembly and expansion of the secretory machinery. Mice globally lacking XBP1 expression except in the liver die soon after birth due primarily to failure of exocrine pancreas (50). The acinar cells exhibit a poorly developed ER accompanied by a decrease in the expression of ER chaperone genes required for maturation and assembly of proteins. Additionally, a significant proportion of these XBP1-deficient cells undergo apoptosis. Overall, it is suggested that XBP1 is critical to balance secretory load and capacity and is necessary for the proper assembly of the secretory machinery (50). A similar conclusion was observed in an inducible *Xbp1* conditional knockout mouse engineered to interrogate the loss of this protein in pancreatic acinar cells (60). These XBP1-deficient cells displayed a dilated ER with disorganized cisternae and a dramatic loss in ZG accumulation (60). ATF4 is also important for pancreatic acinar cell development and viability (61). Mice deficient for ATF4 have severely underdeveloped exocrine pancreata, with significant reductions in the numbers and size of acinar cells, accompanied by significantly lower ZG content and expanded extracellular space and adjacent centroacinar ducts (61).

The basic helix-loop-helix transcription factor MIST1 serves as a TF^Scaling factor^ (29), and is expressed solely in secretory cells including pancreatic acinar cells (62, 63), as well as zymogenic cells of the stomach (64, 65), intestinal Paneth cells (66), plasma cells (67), salivary gland acinar cells (68, 69), and mammary epithelial cells (70). MIST1 augments transcriptional activation or repression of target genes, many of which are critical for the development of the secretory apparatus in these specialized cells (31, 71, 72, 73). Illustrating this idea, PTF1 induces *Mist1* gene expression and, in turn, PTF1 and MIST1 co-regulate the expression of over 100 downstream acinar genes (30). Within this context, MIST1 helps to govern the magnitude of PTF1-directed expression of two broad groups of genes: (i) pancreatic acinar cell-specific genes and (ii) those genes more widely expressed in other professional secretory cells (30). Secretory enzymes and cofactors are part of the restricted gene group, whereas the more broadly expressed genes include proteins involved in secretory protein production, protein folding and processing, and those genes that participate in cellular stress response (30). MIST1 co-regulation of PTF1-directed pancreatic acinar gene expression is not dependent on a stable physical interaction between these two transcription factors, although they frequently bind in proximity in pancreatic acinar cell chromatin (30). In an analogous fashion, MIST1 also co-regulates XBP1-directed expression (31). Upon activation of ER stress, XBP1 induces *Mist1* gene expression (31). Subsequently, MIST1 and XBP1 co-regulate target genes that contain adjacent MIST1 and XBP1 binding sites. These gene targets include those that regulate protein synthesis, processing, transport, and exocytosis networks (31). The details of these functional interactions between MIST1 and these lineage-directing and ISR/UPR transcription factors are not yet clear. Nevertheless, while MIST1 is critical for secretory activity and functions as a potent ER stress-inducible transcriptional regulator (31), it is not considered an integral part of the ISR/UPR but rather serves to scale up gene expression.

### Antibody-secreting plasma cells

The differentiation of B cells into antibody-secreting plasma cells also features a similar three-part program (Fig. 3*B*). Antibodies are synthesized and secreted by plasmablasts and plasma cells, collectively called antibody-secreting cells (ASCs) (74, 75). Early in the immune response, activated B cells develop into large numbers of plasmablasts, which are short-lived, dividing ASCs that have migratory potential and can further develop into mature, terminally differentiated plasma cells (74, 75). The transition from B cell to ASC requires the coordinated change in the expression of hundreds of genes that includes the silencing of key transcription factors that enhance B cell identity, along with activation of ACS-specific regulatory proteins (74, 75, 76). The key transcription factors in the three-part program required for this ASC transition from B cell include the TF^Lineage^ IRF4, the TF^Lineage^ BLIMP1, the TF^ISR/UPR^ XBP1, and the TF^Scaling factor^ MIST1 (74, 75, 76, 77, 78).

IRF4 is essential for regulating immunoglobulin class switch recombination in B cells, for the generation of germinal center B cells, and for the early steps in ASC differentiation and survival (79, 80, 81, 82). IRF4 belongs to the Interferon Regulatory Factors family of trans-acting proteins that control several functions, including the differentiation and development of hematopoietic cells and the control of host defense against pathogens (83, 84, 85). IRF4 contains a helix-loop-helix motif that mediates DNA binding (83, 86, 87, 88) and a C-terminal IRF association domain (IAD) that mediates both homo- and heterodimeric protein-protein interactions with various transcription factors (83, 87). As IRF4 expression increases in the B-cell, IRF4 homodimers are favored that bind to interferon sequence response elements (ISREs) found in genes that drive plasma cell differentiation (89, 90).

A critical IRF4-target gene in ASC differentiation is *Prdm1*, which encodes the protein BLIMP1 (89, 90). BLIMP1 contributes to the silencing of genes by recruiting co-repressors such as histone deacetylases (91), Groucho co-repressors (92), lysine-specific demethylase 1 (LSD1) (93), and histone H3 methyltransferase G9a (94). BLIMP1 also contributes to the induced transcription of selected genes that are important for ASC differentiation. For example, in mouse plasmablasts, BLIMP1 induces the expression of UPR genes and indirectly activates *Xbp1* expression while repressing the expression of key B cell genes including *Id3*, *Bcl6* and *Pax5* (95, 96, 97). Although ASC differentiation can proceed without XBP1, the absence of this UPR protein impairs ER biogenesis, reduces expression of secretory pathway genes, decreases cell size, and limits overall protein synthesis (4, 98, 99, 100, 101, 102, 103). Mice with a B cell–specific deletion of *Xbp1* maintain normal ASC numbers in both resting and immunized conditions. However, these XBP1-deficient ASCs show markedly reduced antibody secretion and fail to acquire the characteristic morphology of antibody-secreting cells (100). Supporting these data, retroviral overexpression of XBP1 in various B cell and non–B cell lines induce a secretory phenotype (4). For instance, forced XBP1 expression in the Raji (human) and WEHI-231 (mouse) B cell lines leads to upregulation of multiple ER and secretory pathway genes (4). Fluorescent staining of Raji cells with BFA-BODIPY, which labels the ER and Golgi, revealed a 2- to 3-fold increase in fluorescence intensity following XBP1 overexpression. A similar enhancement was observed in the human kidney cell line 293, indicating that XBP1 can drive a secretory gene expression program independently of cell type (4).

It is interesting that splicing of *Xbp1* mRNA is not suggested to be increased until the later stages of ASC differentiation (23, 103). This observation suggests that overt ER stress is not required to drive early differentiation processes. Rather, the high levels of *Xbp1* mRNA may be sufficient for expression of spliced XBP1 protein and its target gene transcription even when there are only modest amounts of ER stress and low IRE1 endonuclease activity that helps drive splicing of *Xbp1* mRNA. In this "hypersensitivity" model, the modest ER stress during early differentiation would trigger significant XBP1 protein transcriptional activity without robust activation of the UPR sensor proteins. This model would help explain the observation that the other UPR sensory proteins ATF6 and PERK are largely dispensable for differentiation of ASCs (23, 104, 105).

A major gene target of XBP1 is MIST1, the TF^Scaling factor^ that was discussed earlier. Expression of MIST1 is highly induced by the late stages of plasma cell differentiation (78). Induced MIST1 expression that occurs during ASC differentiation is suggested to lower expression of BLIMP1, contributing to an increase of BLIMP1-repressed target genes (78). In the absence of MIST1, there is enhanced BLIMP1 and the number of plasma cells are reduced although each cell secretes more antibody, suggesting that MIST1 restricts antibody secretion *via* its suppression of BLIMP1 (78). Therefore, there are multiple points of integration between the UPR, lineage-specific and scaling factors that are critical for setting the secretory capacity during B cell differentiation.

### Pro-opiomelanocortin (POMC) pituitary secretory cells

Pro-opiomelanocortin (POMC) pituitary secretory cells have a very high demand for producing and releasing corticotropes from the anterior lobe, and melanotropes from the intermediate lobe (106). Development of these mature cells is driven by the transcription factors TPIT, XBP1, and CREB3L2 (22) (Fig. 3*C*). TPIT (T-box transcription factor) is a member of the highly conserved T-box family of transcription factors characterized by a DNA-binding domain termed the T-BOX (107, 108) that is critical for many developmental processes, including embryonic, organogenic, and limb development (109). In the binary model of pituitary cell differentiation, the cortico/melanotroph (ATCH, αMSH) and gonadotroph (FSH, LH) lineages arise from a common precursor (110). In the cortico/melano/gonadotroph lineage, expression of and antagonism between TPIT and the orphan nuclear receptor steroidogenic factor 1 (SF-1), establishes the POMC and gonadotroph lineages, respectively (110). The TF^Lineage^ TPIT drives terminal differentiation of the POMC-secreting cells (110, 111, 112). During the early stage of differentiation, TPIT activates both UPR proteins XBP1 and CREB3L2 (22). The TF^ISR/UPR^ XBP1 triggers the physiological UPR and stimulates ER biogenesis and expansion of the secretory pathway, while CREB3L2 functions primarily as a TF^Scaling factor^ in pituitary cell differentiation (22) (Fig. 3*C*). CREB3L2 furthers robust expression of genes involved in ribosome biogenesis and translational control and therefore functions to enhance the protein synthesis capacity early in differentiation (22).

### Secretory osteoblasts

Osteoblasts are professional secretory cells. Morphologically, osteoblasts are readily identified on bone surfaces by their copious volume of endoplasmic reticulum (ER) and Golgi, allowing them to be high-capacity secretors of bone matrix—their primary function (113, 114, 115). Like other professional secretory cells, the osteogenic differentiation of skeletal stem cells can be cast into the three-part program (Fig. 3*D*). The TF^Lineage^ for the secretory osteoblast include RUNX2 and SP7 (OSTERIX) (116, 117, 118). RUNX2, a member of the Runx family of transcriptional regulators, forms a heterodimer with Core-binding factor beta (CBFβ) to drive osteoblast-lineage commitment in mesenchymal stem/progenitor cells (MSPCs), also known as bone marrow stromal cells (BMSCs) (119, 120, 121, 122, 123, 124). RUNX2 induces the expression of SP7 (118, 125), a member of the Sp/XKLF (specificity protein/Krüppel-like factor) family of transcription factors (126, 127) that couples with distal-less homeobox (Dlx) factors, such as Dlx5 and Dlx6, to direct multiple genes that support the osteoblast differentiation (128). The RUNX2/SP7-positive osteoprogenitors differentiate into pre-osteoblasts and in turn SP7 drives the differentiation of pre-osteoblasts to secretory osteoblasts (129, 130). RUNX2/SP7 also drive the expression of bone matrix proteins, such as collagen and osteocalcin (129, 130, 131).

After the secretory osteoblast has delivered the bone matrix there are three possible fates: it can remain on the bone surface and become a quiescent bone lining cell, it may undergo apoptosis, or the cells may become trapped within its own matrix and differentiate into an osteocyte (132). SP7 is critical for the osteoblast-to-osteocyte transition (133). These terminally differentiated cells are the most abundant cells in the skeleton, are long-lived, and act as hormonal and mechanical sensors *via* their long, neuron-like dendritic processes (134, 135, 136). SP7 is required for the formation of these processes (133) and the osteocyte network plays a critical role in maintaining bone homeostasis and in regulating skeletal response to hormones and mechanical loading (132, 137, 138).

Many of the ISR/UPR regulators are activated and required for osteoblast differentiation (35). For example, the UPR protein OASIS plays significant roles in osteoblast differentiation and bone formation (139, 140, 141, 142). Mice deficient for OASIS have severe osteopenia resulting from a decrease in osteoblast secretion of type I collagen (139). OASIS is required for the later stages of osteoblast differentiation but does not appear to have a significant impact on the very early stages of osteoblast commitment (139). During osteogenic differentiation, OASIS undergoes RIP, and the N-terminal polypeptide moves to the nucleus to drive *Col1a1* gene expression through direct binding to promoter regions of this gene. Indeed, immunohistochemistry demonstrated that osteocalcin and procollagen accumulated in the distended rough ER of *Oasis*^*−/−*^ osteoblasts (139).

The UPR sensor, PERK, also plays a central role in bone development. PERK-deficient mice develop severe osteopenia soon after birth and exhibit decreased mineralization of the bone matrix (143), consistent with the observations that in culture PERK-deficient osteoblasts show a delay in mineralization (143, 144, 145). ATF4, a key transcription factor downstream of PERK, plays a critical role in osteoblast differentiation and mineralization (144). Mice lacking ATF4 (*Atf4*^*−/−*^) exhibit osteopenia and significantly reduced collagen synthesis by osteoblasts (146). In calvarial osteoblasts from *Perk*^*−/−*^ mice, ATF4 expression is markedly diminished, accompanied by reduced alkaline phosphatase activity and delayed mineralization in culture compared to wild-type cells (144). Notably, reintroduction of ATF4 into these knockout cells restores their differentiation potential (144). Likewise, silencing ATF4 using siRNA impairs differentiation of the osteoblast-like MC3T3-E1 cell line (145). Additionally, XBP1 is required for the development of the secretory osteoblast *via* its activation of SP7 expression (147), which drives the latter stages of osteoblast differentiation (129, 130). Finally, the master transcription factor for osteoblast differentiation, RUNX2, upregulates the UPR protein ATF6, which in turn contributes to osteoblast osteocalcin expression (148), the most abundant non-collagenous protein in bone and required for optimal bone strength (149). Therefore, multiple ISR/UPR transcription factors are integrated in the osteogenic processes.

Nuclear Matrix Protein 4 (NMP4, ZNF384 [human], ZFP384 [mouse]) is suggested to function as a TF^Scaling factor^ in osteoprogenitor cells by dampening the magnitude of induced ISR/UPR gene expression (150, 151). While mice deficient for NMP4 alleles have no baseline phenotype, given an osteoanabolic drug they form 2 to 3 times as much bone as their equally treated wild-type littermates (152, 153, 154). While *Nmp4* appears to be expressed in all tissues (155), the enhanced response to osteoanabolics is largely driven by MSPCs/osteoprotgenitors (154). Indeed, conditional loss of *Nmp4* in osteoprogenitors, but not mature osteoblasts or osteocytes duplicates the enhanced parathyroid hormone (PTH) and sclerostin antibody (Scl-mAb)-induced bone formation observed in global *Nmp4*^*−/−*^ mice (152, 153). *Nmp4*^*−/−*^ MSPCs isolated from experimental mice exhibited accelerated mineralization and developed into super-secretory osteoblasts that showed increased ribosome biogenesis, increased ribosomes and protein synthesis, and greater collagen protein secretion compared to *Nmp4*^*+/+*^ cells (150, 151, 156). Loss of NMP4 significantly elevated glycolysis in MSPCs (150), the major metabolic pathway to meet ATP demand during osteoblast differentiation (157).

The mechanistic basis for how NMP4 functions as a scaling factor is emerging. NMP4 is a Cys_2_His_2_ zinc finger transcription factor (155) and a scaffold/matrix attachment region-binding protein (S/MARBP) (158, 159, 160). S/MARs are sequences of DNA, typically AT-rich, that tether chromatin to the nuclear matrix of the cell (159, 161, 162). S/MARBPs, including NMP4, integrate chromatin organization and accessibility with the regulation of gene expression (159, 163, 164, 165). NMP4 supports chromatin looping, which brings distal enhancers and their promoters into proximity (164, 165, 166, 167, 168, 169, 170). ChIP-Seq identified over 15,000 NMP4 binding sites throughout the osteoblast genome with a large percentage near gene promoters (156) and in proximity to chromatin loop-forming proteins, such as CTCF (156, 159, 164, 165). In this way, NMP4 is suggested to organize chromatin to lower the expression of ISR/UPR genes and related protein synthesis and secretory processes. The loss of *Nmp4* leads to sharply elevated expression of many ISR/UPR-related genes, including *Oasis*, *Atf4*, *Ire1,* and *Xbp1* genes (150, 171). The opposing function of *Nmp4* and *Oasis* gene expression may underlie the different phenotypes of *Oasis*^*−/−*^ and *Nmp4*^*−/−*^ mice. As previously stated, the loss of *Oasis* leads to osteopenia, and isolated osteoblasts exhibit diminished collagen expression and mineralization (139). *Nmp4*^*−/−*^ mice, by contrast, show enhanced bone material properties and the osteoblasts exhibit accelerated mineralization and increased collagen secretion (150, 156).

NMP4 resembles the TF^Scaling factors^ MIST1 and CREB3L2, but with some key differences. All three transcription factors appear to be associated with critical steps in protein secretion. Whereas MIST1 is suggested to be uniformly associated with nearly every step of protein production and delivery in the pancreatic acinar cell (30), the role of CREB3L2 as a scaling factor is restricted to expression of genes involved in protein synthesis in the POMC-secretory cells (22). The scope of NMP4 function is more analogous to that of MIST1 in that its loss impacts selected processes in protein production and delivery. Interestingly, MIST1 and CREB3L2 appear to largely enhance the activities of target genes in the secretory pathways (22, 30), whereas NMP4 primarily limits gene activity in osteoblasts (150, 151, 156). The elevated protein production through the ISR, increased glycolysis to support this production, and the enhanced protein processing *via* the UPR are suggested to be key to the development of the *Nmp4*^*−/−*^ super-secreting osteoblast. These functional relationships and hierarchies between NMP4 and the key ISR/UPR sensors and their target genes during osteoblast differentiation remain to be fully clarified.

## Does the ISR/UPR limit osteoanabolic therapy?

Osteoporosis is a disease of reduced bone mass leading to low energy bone breaks or fragility fractures (172). In the United States, 55% of people over 50 years of age are osteoporotic (173). The etiology of osteoporosis is multifactorial, and the major risk factors include low peak bone mass, genetics, aging, hormonal factors, smoking, physical inactivity, and various medications (174, 175). Simply put, osteoporosis is a disease of imbalanced bone remodeling (176). Bone is constantly turned over throughout life, about 10% per year, replacing old with new (177, 178). Synthesis of new bone to replace the old is central for mechanical integrity and to maintain serum calcium/phosphate balance in the bloodstream (179). Osteoclasts resorb bone and mature osteoblasts secrete bone matrix. The process of bone remodeling is normally balanced but when resorption outpaces formation, osteoporosis can result.

Two types of drugs are used to bring remodeling into balance. Anti-resorptives target osteoclasts to slow or stop resorption. Osteoanabolic drugs target the osteoblast lineage and enhance bone matrix secretion, *i.e.,* bone formation (180). There are presently three osteoanabolics approved by the U.S. FDA for severe osteoporosis. These drugs include an analogue to PTH (PTH 1-34, known as teriparatide), an analog to parathyroid hormone-related peptide (PTHrp 1-34, known as abaloparatide), and romosozumab, an antibody to sclerostin (Scl-mAb) (181, 182, 183, 184). These osteoanabolic drugs leverage distinct molecular pathways and mechanisms of bone turnover for skeletal gain (185, 186, 187, 188, 189). However, despite these significant differences, all three of these drugs lose efficacy after about 2 years (190). This relatively brief interval of high potency limits their utility for treating a chronic degenerative disease like osteoporosis (190, 191).

**Figure 4:**
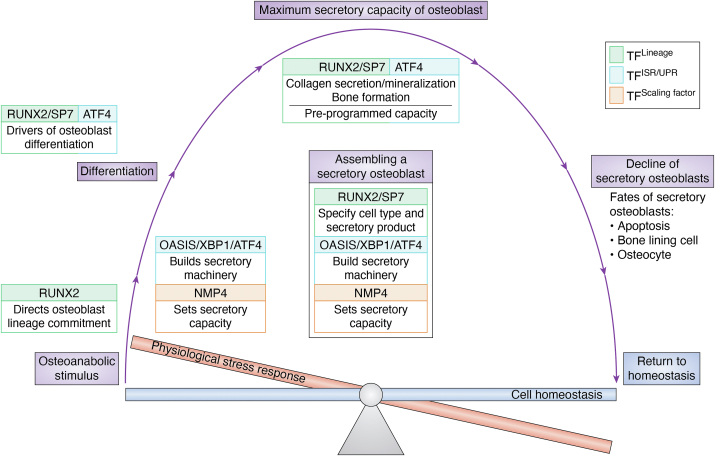
**Reframing the osteoblast secretory capacity within the context of the three-part transcriptional program for assembling the secretory osteoblast.** In this model, osteoanabolic drugs trigger the differentiation of bone marrow mesenchymal stem/progenitor cells. One limb of this three-part program is driven by the transcription factor RUNX2, which commits the cell to the osteogenic lineage. RUNX2 also stimulates the expression of SP7 and the RUNX2/SP7-positive osteoprogenitors differentiate into pre-osteoblasts and in turn SP7 drives the differentiation of pre-osteoblasts to secretory osteoblasts. The second part of this program assembles the massive protein synthesis and processing machinery necessary for the mature secretory osteoblast. ISR/UPR sensors, *e.g.* OASIS, and their downstream effectors, *e.g.*, XBP1 and ATF4, drive the assembly of the protein production and secretion machinery. ATF4 multitasks and not only reshapes the translatome of the nascent secretory cell but is also a driver of osteoblast differentiation. The third part of the program is driven by NMP4, which acts as a scaling factor to modulate the bone matrix delivery capacity of the mature osteoblast. This three-part program establishes the osteoblast secretory capacity and therefore the potency of any osteoanabolic drug. Improving drug efficacy requires changes in this developmental program, *e.g.*, silencing NMP4, before the cell becomes secretory.

Does the limit to the potency of any osteoanabolic drug reflect the constraints of the mature osteoblast secretory capacity and is this ceiling set in the skeletal stem cell before the cell becomes secretory? The origin as well as the capacity of the osteoblast’s response to these drugs may be derived from the three-part transcriptional program in the osteoprogenitor (Fig. 4). In this model, osteoanabolic drugs trigger the differentiation of bone marrow mesenchymal stem/progenitor cells (192), and the development of the professional secretory osteoblast progresses as described earlier. This three-part program establishes the secretory capacity and therefore the potency of any osteoanabolic drug before the osteoblast becomes secretory. Improving drug efficacy is suggested to require changes in this developmental program, such as silencing NMP4, *before* the cell becomes secretory. Targeting ISR/UPR scaling factors early in osteoblast development may enhance the therapeutic efficacy of these osteoanabolics.

Does this high-capacity *Nmp4*^*−/−*^ physiological ISR/UPR forestall “proteostasis collapse” and promote healthy aging? Does *Nmp4*^*−/−*^ bone maintain the enhanced response to osteoanabolic drugs well into old age? Proteostasis collapse is the decline in the ability of aging cells to induce stress response pathways (193, 194, 195). One of the central features of aging is the deterioration of proteostasis, where the function of the ISR/UPR has declined (193, 195, 196). Studies in human tissues, along with multiple models, demonstrate that adaptive UPR signaling promotes healthy aging (196, 197, 198, 199). Strategies to improve ER proteostasis using small molecules and gene therapy reduce the age-induced decline of organ function in mammals (196). The rescue of the IRE1-XBP1 and PERK, along with protein-folding chaperones, has beneficial effects on age-associated deterioration of cell and organ homeostasis (198, 200, 201, 202).

## Summary and conclusions

The concept of the physiological ISR/UPR has led to the re-evaluation of what is meant by cellular stress (22, 25, 33, 203). The ISR/UPR participation in secretory cell differentiation places this program in a very different role than maintaining homeostasis but instead this program drives dramatic changes that require temporal disequilibrium. Also emerging from studies on secretory cell differentiation is that a relatively small number of transcription factors control distinct aspects of this process, which include (i) lineage commitment and specification of the secretory products, (ii) the building of the protein production and secretory machinery, and (iii) setting the capacity of this machinery. Scaling factors can be part of the ISR/UPR or be distinct from this cast of proteins. These scaling factors establish secretory capacity early in the cell’s development. This shift in paradigm may be clinically significant. Targeting scaling factors in immature secretory cells may enable reprogramming of the capacity of mature tissues to better respond to drug therapies. This approach could also offer a strategy for rejuvenating aged cells, tissues, and organs, making them more receptive to treatment.

## Data availability

All data presented are contained within the manuscript

## Conflict of interest

The authors declare the following financial interests/personal relationships, which may be considered as potential competing interests:

R. C. W. is a member of the advisory board of HiberCell Inc. The other authors declare that they have no competing interests.
